# The role and interplay of institutions in water governance in the Central Rift Valley of Ethiopia

**DOI:** 10.12688/f1000research.138939.1

**Published:** 2023-11-06

**Authors:** Endalew Jibat, Feyera Senbeta, Tesfaye Zeleke, Fitsum Hagos

**Affiliations:** 1 College of Development Studies, Addis Ababa University, Addis Ababa, Ethiopia; 2 International Water Management Institute, East Africa Office, C/o ILRI-Ethiopia campus, Addis Ababa, Ethiopia

**Keywords:** Institutions, Governance, Role of Institution, Institutional Interplay, Water Users

## Abstract

**Background:**

Institutions can play a key role in coordinating how natural resources are effectively managed and used without over-exploitation. Institutions are laws, policies, and organizational arrangements that permit, forbid or regulate human action. This study aimed to look into the roles of formal and informal institutions, and their interactions in water resources governance in the Central Rift Valley (CRV), Ethiopia.

**Methods:**

Key informant interviews, focus group discussions, and secondary data sources were employed to collect relevant data.

**Results:**

The result of the study indicated that the influence of informal institutions on formal institutions or vice versa was insignificant, and unable to change the actions of water users in the CRV. Other limitations observed in water resources governance in the CRV include a lack of actors’ clear roles and responsibilities, absence of meaningful decentralization, limited engagement of key actors in policy development, lack of synergy between the institutions, and absence of enforcement mechanisms.

**Conclusion:**

Considering the local contexts and community’s traditional knowledge of water governance in water-related policy, rules, and regulations, and enhancing the capacity of local-level institutions, strong interplay among all institutions involved in water governance, and meaningful actors’ engagement were recommended to advance the role of institutions in water resources governance in the CRV and in the country.

## Introduction

Institutions can shape natural resource users' actions and encourage efficient resource use ( Medase *et al.* 2023). The term ‘institution’ is referred to multiple times in development literature such as specific organizations, human relationships in a society, and rules that individuals use to shape specific relationships with others ( Pradhan *et al.* 2022). Institutions are also laws, policies, and organizational arrangements that societies develop to allow, prohibit, or regulate certain human actions ( Chopra and Ramachandran 2021).

Water management is a mix of formal and informal institutions in many countries in the world ( Yeboah-Assiamah *et al.* 2017). Formal institutions are related to the official channels of the governmental system, and enforced by lawful procedures, while informal institutions refer to socially shared rules such as social or cultural norms and reflect local people’s views ( Gilmore *et al.* 2022). Both formal and informal institutions are shared, enforced, and long-lived ( Hassenforder and Barone 2019). Empirical studies, e.g., Yami *et al.* (2009) indicate that both formal and informal institutions played a great role in natural resources governance.

Institutional diversity can enhance the capacity of a certain community by educating multiple ways of responding to challenges ( Pellowe and Leslie 2020). For instance, Yami *et al.* (2009) revealed that informal institutions contributed to sustainable Common Pool Resources (CPR) management in Sub-Saharan Africa through common decisions, reducing costs for CPR users, taking advantage of local knowledge, and implementing locally agreed rules. Institutional measures such as enforced formal laws, informal norms, and standards shared among communities help to govern the individual actions of resource users ( Medase *et al.* 2023).

Several studies indicate that the interaction of formal and informal institutions results in positive effects on natural resources governance. For instance, Yeboah-Assiamah *et al.* (2017) reported that informal institutions were harmonized with formal and synergistically improved the viability of the natural resources in Ghana. Similarly, both formal and informal institutions have shaped fishing practices in Mexico ( Pellowe and Leslie 2020). In some cases, informal institutions gradually become part of formal institutions, and formal also take informal forms in governing resources such as water resources ( Nhundu *et al.* 2015). Hence, scholars, for instance, Tinoco (2012) suggested the need to consider both formal and informal water institutions to operate and enforce them at different levels based on local reality.

Productive interactions between formal and informal institutions could be facilitated when shared experiences exist between diverse actors. Mechanisms designed to enable shared learning, feedback, and adaptation experiences between both institutions can facilitate positive management development, and encourage institutional interplay (June *et al.* 2019). It is also important to devise intervention mechanisms to enhance the capacity of the informal systems to manage water resources because both institutions can draw lessons from each other ( Nhundu *et al.* 2015).

On the other hand, there is also a situation where formal and informal institutional conflicts have notably compromised water service delivery and water security ( Muok and Onyango 2020), as a result of poor planning, unable to align national and regional strategies, and political intervention. In a similar manner, the informal institution of the ancient caste system in India hindered the effectiveness of formal institutions ( Haider 2017).

The effectiveness of institutions depends on the nature of institutions and their enforcement mechanisms ( Yeboah-Assiamah *et al.* 2021). The strength of the governance system, planning, and financial adequacy can be factors affecting the effectiveness of institutions and are essential for sound water governance ( Tortajada 2010b). Institutional ineffectiveness may be caused because of inadequate consideration of the human element and its failure to take into account the need for awareness of future uncertainty to adapt to changes ( Akamani 2016).

In this paper, institutions are defined as formal and informal institutions, which concern what and how actors are playing their roles in water resources governance actions. Formal institutions include rules and regulations decreed to use and conserve water resources in the country, the role of state structures engaged in water decisions and actions, the contribution of development partners and irrigation water user associations in water resources governance practices in the Central Rift Valley (CRV) of Ethiopia. Informal institutions include traditions and customs practiced in water uses and conservation activities in the CRV of Ethiopia. The interplay of institutions is the interaction of these institutions (whether they overlap/are similar or conflict) on the grassroots level in irrigation water resources collective actions in the study area. Hence, the main aim of this study was to assess the roles of formal and informal institutions and their interplay in water resources governance in the CRV of Ethiopia.

## Methods

### Ethical approval and consent

The ethical clearance letter of this study No/0035/2023 was signed by Teshome Tefesse (PhD), Chairperson of the institutional review board of the College of Development Studies at Addis Ababa University, Ethiopia. The proposal was defended in February 2021, and during that time providing an ethical approval letter was not started at the College. The proposal for the research was reviewed and approved by the committee of the institutional review board. Written informed consent was also obtained from all participants who allowed the publishing and publicizing of the data. During data collection, participants were informed that the data would be used for the PhD dissertation of the corresponding author and could be published.

### Study area

**Figure 1.**
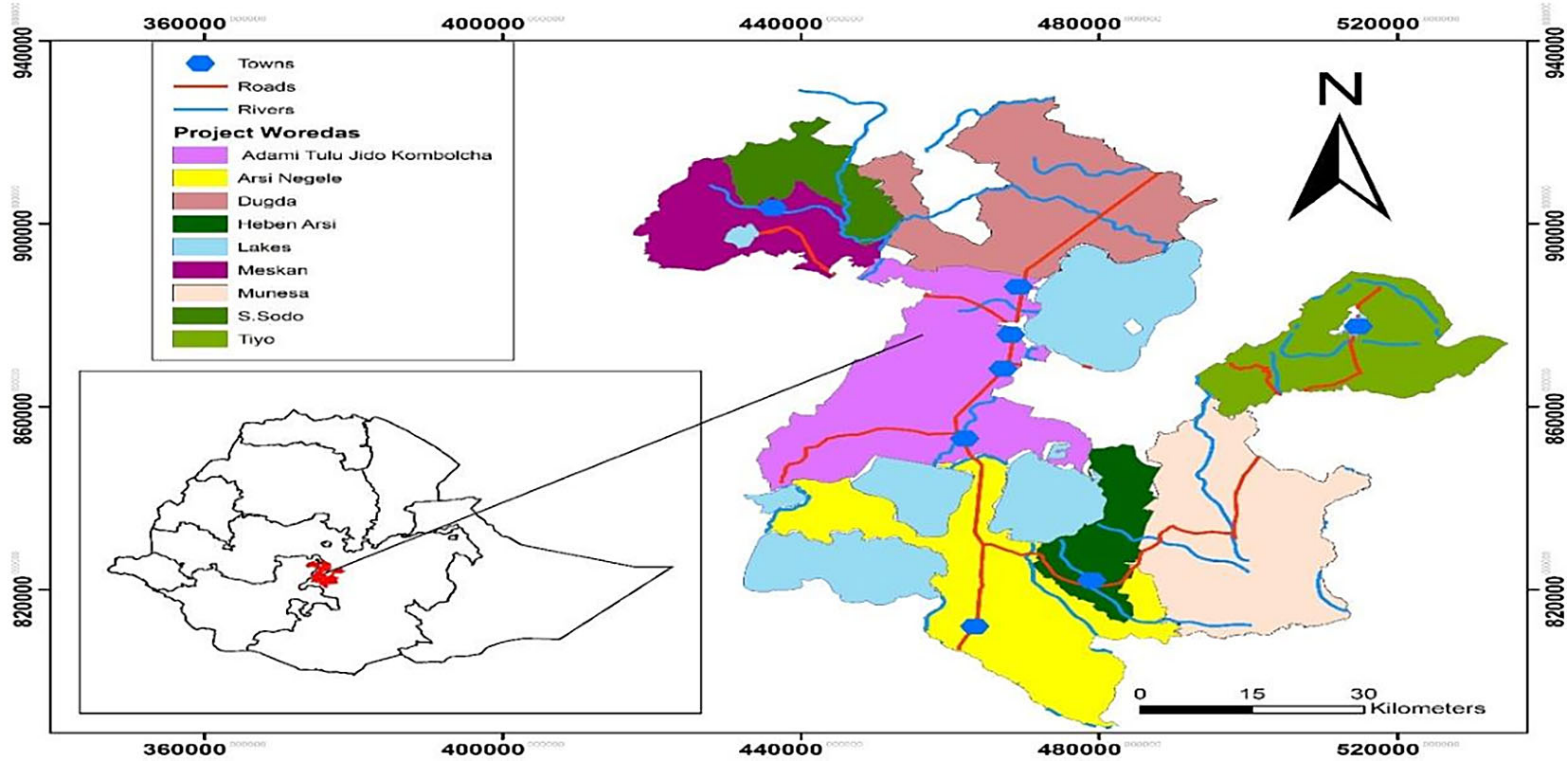
Location map of the study area (Developed 2020).

The Central Rift Valley (CRV) of Ethiopia is part of the African Great Rift Valley, and it is located between 38°00’-39°30’ east longitude and 7°00’- 8°30’ north latitude ( Pascual-Ferrer and Candela 2015). The altitude of the CRV ranges from 1500m – 4000m above sea level. The climate is semi-arid, and the mean annual temperature is around 20°C in the valley. The rainfall in the CRV is erratic and high rainfall intensity and extreme spatial and temporal variability were the experiences of the area ( Pascual-Ferrer and Candela 2015). The average annual rainfall ranges from 650 mm on the rift floor to 1150mm in the highlands ( Tafesse Matewos & Tewodros Tefera 2019). The study area ( Figure 1) covered large areas and was found in two different administrative boundaries, Oromia Regional State and South Nation Nationalities and Peoples Region.

The majority of the population relied on subsistence farming and small landholding size ( Vilalta 2010). The major farming system in the CRV was a small mixed rain-fed production system consisting of grain crops and livestock farming. The main livestock productions in CRV were cattle, sheep, and goats; and donkeys, mules, and horses were kept for transportation ( Vilalta 2010).

Food security issues and access to health facilities were the major concern in CRV, and there were few opportunities to engage in income-generating activities ( Vilalta 2010). During the disaster and stress period, cultivators developed several survival strategies such as seasonal out-migration, engagement in off-farm activities, leasing out of farmland, and selling livestock.

The main source of irrigation was from surface water (44% is by river diversion and 31% from Lake Dembel/Ziway), and 25% was from groundwater ( Pascual-Ferrer and Candela 2015). Agricultural production and its related activities were the main sources of the economy. About 67% of the CRV's Gross Domestic Product (GDP) was from agriculture, which includes crops, livestock, fisheries, and forestry while industry and service sectors accounted for 10% and 23% respectively ( Pascual-Ferrer and Candela 2015).

### Research design

This study employed a survey design and qualitative research approach. A survey is any activity that collects information in an organized and methodological manner from some or all units of a population using well-defined methods and procedures ( Creswell 2014). A survey has benefits including generalizing from a sample to a population. The rationales for selecting a sample survey for this study were cost implication and timeliness to produce the result of the report.

### Sampling technique and method of data collection

The study employed a multi-stage sampling technique. The study covered wider areas (both site and beyond the site). In this study, the CRV was categorized into three catchments (upper, middle, and lower catchments). From each of the categories, one Woreda was selected using a simple random sample method. From each selected Woreda, two Kebeles were purposely selected based on long-term experiences of irrigation activities to get sufficient data for the study. Accordingly, East Meskan, Dugda, and Adami Tullu Jiddo Kombolcha were the selected Woredas from the upper, middle, and lower catchments respectively. The sampling frame included individual farmers or households, who are economically active (from age 18-64) and involved in cultivating crop/vegetable/fruit and livestock production.

The study used key informants’ interviews, focus group discussions, and a review of policy documents and literature to collect relevant data. The study involved various government tiers: federal, regional, District, and Kebele (which is the smallest administrative unit in the country). A total of 36 key informants were interviewed. Key informants were selected from government organizations, Irrigation Water Users Associations (IWUAs), fishery associations, farmers, local elders, development partners, researchers, and representatives of informal institutions (from age 18-64). Semi-structured guiding questions were prepared for data collection. The interview guide was prepared and discussed in detail with supervisors before field data collection.

In addition, four focus group discussions (FGD) were held. The participants were selected from farmers, IWUAs members and local elders considering gender, age, occupation, experiences on irrigation activities, and accessibility to information. The total participants of focus group discussion were 28 people; 8 people in each group of 1 and 2, and 6 people in each group of 3 and 4. Participants were categorized based on the selection criteria mentioned in this paragraph. Five main guiding questions were prepared, and explained by moderator for the participants.

The data was collected at the workplace, and on field through face-to-face personal contact interviews and group discussions, and only the researcher and participants were presented. The data collection was assisted by audio recording based on the full consent of participants. The field note was made during the interview and focus group discussion and was revised using the audio recorded. The start and end time of the interview and focus group discussion duration was recorded. Key informant interviews and focus group discussion activities were conducted until data saturation was reached. The data collection was carried out using local languages (Afan Oromo and Amharic) and translated to English later on. The data were transcribed, organized, and used for analysis. Data were collected in March and May 2021.

Regarding secondary data, different laws and water policy-related documents including the Constitution of the Federal Democratic Republic of Ethiopia ( FDRE 1995), Environmental Policy of Ethiopia ( FDRE 1997), Water Resources Management Proclamation ( FDRE 2000), Water Resources Management Regulation ( FDRE 2005), Ethiopian Water Sector Strategy ( FDRE 2001), Power and Duty of Ministry of Water and Energy, and Power and Duty of Ministry of Agriculture ( FDRE 2018a), Irrigation Water Users Association ( FDRE 2014), Power and Duty of the Basin Development Authority ( FDRE 2018b), National Water Policy and Strategy ( FDRE 2020), and other related policy documents were analyzed.

### Data organization and method of analysis

Data collected through interviews and focus group discussions were transcribed and documented using Word documents. The transcribed data were coded and organized under sub-themes of the study by carefully reading the transcribed data. The backgrounds of the respondents were organized separately using Excel Sheets. Following data organization; the data were imported into NVivo 11 software for coding and analysis purposes. In a similar manner, different water policies and strategies, proclamations, regulations, and related PDF documents were imported into NVivo 11.

After importing all the necessary information into the NVivo 11 software application major themes and sub-themes were created using the software. As Zamawe (2015) stated, NVivo helps in the analysis of qualitative data by facilitating the tasks of managing data and ideas, modeling visually, and reporting. Following this procedure, all the imported data were carefully read and coded under each of the major themes and sub-themes, and qualitative analysis was carried out using a thematic approach. The study was organized and analyzed under major themes such as the role of formal and informal institutions, and the interplay between institutions.

## Results

### Institutional arrangements

Federal and two regional governments were engaged in managing water resources in the CRV of Ethiopia. At the federal level, various organizations such as the Ministry of Water and Energy, Ministry of Agriculture, Ministry of Irrigation and Lowland Development, and Rift Valley Basin Development Office were established and involved in water resources governance in the study area (FDRE Proc. No. 1097/2018; FDRE Proc. No. 841/2014). The two regional states have also arranged similar water-related organizations, and engaged in various activities related to water resources governance including at the District level. In addition, at the Kebele level (the lowest administrative unit in Ethiopia) Development Agents (DAs) and other natural resources experts were assigned to support the development by providing extension services and advice on crop and livestock production, and natural resources conservation activities including water resources.

The federal Constitution (Art 52/3 (d)) granted regional governments to administer natural resources in accordance with federal laws. The existing institutional arrangements indicate that agencies established at regional and lower levels were engaged in water resources governance activities in the area. The Constitutional provision promotes decentralization of authority to the lower administrative levels. However, evidence from this study indicates that there was a lack of alignment between the constitutional provisions and the implementation of rules and regulations on the ground. For instance, one of the key informants (KI-1) mentioned that:

“ *Alignment between Constitutional provisions and implementation are lacking when going down from federal to region, District, and Kebele levels. Integration is lacking among respective sectors; the agriculture sector is not worrying about water efficiency and buffer zone areas while doing irrigation activities; the industry sector is not worrying about water pollution. Water sectors were not integrated (they have no common plan), and each of the institutions ran their duties independently without considering water saving and conservation contrary to the rules and regulations.*”

Institutional arrangements were established at all levels; however, their coordination and integration were weak to improve water resources governance due to a lack of enforcement mechanisms of the existing rules and regulations, and a lack of systematized monitoring and evaluation in the study area.

Empowering the local level institutions and decentralization were tried to be implemented in the study area. However, there was a capacity gap among water resource users, particularly farmers and public institutions that are supposed to support and enforce the implementation of improved water governance in the area. One of the key informants (KI-2) stated that “Water sectors do not have equivalent capacity in terms of finance, logistics, knowledge, technology, and human resources. Only roles were decentralized to the lower levels without capacitating them with finance, knowledge, expertise, and other essential resources.” Another key informant (KI-8) also added that clear rules, regulations, directives, and guidelines that enable lower-level administration were not fully decentralized to manage water for sustainable uses in the study area.

Data and information collected for this study show that required institutional arrangements were established and in place at all levels. However, implementation limitations were observed. One of the key informants (KI-29) stated that:

“ *All structures of water sectors exist. However, the institutions were not implementing the policy and regulation of water resources management on the ground. There is no problem regarding structure, but limitations on implementation. This may be caused by a lack of capacity to implement their mandate and a lack of monitoring and evaluation from respective Regional and Federal level water sectors. The local government agencies should be capacitated by required knowledge and resources.*”

In addition to government institutional arrangements, there were also community-based institutions (for instance, Irrigation Water Users Associations/IWUAs) that were engaged in water resources governance in the CRV. The government recognized the role of these institutions in improving water use efficiency and protecting the environment. To strengthen these institutions, the federal government decreed IWUA’s proclamation (FDRE Procl. No. 841/2014, FDRE Regulation 441/2018) with the objectives: to manage and operate an irrigation and drainage system; to provide water equitably to its members for agricultural purposes; to maintain, and rejuvenate and improve the irrigation and drainage system; and to maintain and operate irrigation equipment.

The proclamation also stated that the IWUAs shall be guided by principles of fairness and equity in decision-making and allocation of irrigation water; preventing wastage and pollution of water; protecting and administering irrigation and drainage system within the operation area; and complying with a system of cost recovery and efficient use of resources. The associations have also developed their own rules (agreements) that address how to distribute water for the members, how to undertake scheme maintenance and impose sanctions on non-compliance, and the way they conduct monitoring and evaluation. The performance of the associations varied from association to association due to factors such as financial capacity, the commitment of the IWUAs committee, and support from the district office.

In addition, development partners and NGOs were also involved in water resources matters, particularly in protection and conservation activities in the CRV of Ethiopia. Data obtained from key informants (e.g.KI-19 and KI-26) shows that development partners and NGOs were supporting farmers and IWUAs in awareness creation, material support, and providing capacity building training. An informant added that gap was observed among government institutions and community-based organizations in taking the responsibility of sustaining projects initiated by development partners and NGOs when their projects were phased out.

### Role of public institutions in water governance in the CRV

The government of Ethiopia adopted various water policies, rules, and regulations to improve water resources governance in the country. Information from secondary data ( FDRE 1995; FDRE 2001; FDRE 2005) shows that the policy documents advocate the necessity of building and strengthening the capacity of water institutions, institutional stability, and user-based management of irrigation systems. In addition, the rules and regulations promote the development of appropriate institutional structures as well as decentralization to the lowest level of governance for better and more efficient management of water resources. Various public institutions were also established to play their roles in the implementation of these policies and regulations. For instance, water-related sectors such as the Water and Energy Ministry, Basin Development Office, Ministry of Agriculture, Ministry of Irrigation and Lowland Development, Environment Protection Authority, and others were established at the federal level to effectively manage water resources.

One of the roles of public institutions in improving water resources governance is undertaking studies and collecting relevant and updated water-related information. In this regard, one of the key informant (KI-7) expressed that some of the institutions (e.g. Rift Valley Basin Development Office/RVBDO) have conducted the study, and the study addressed water use sustainability, current and future demand for water, the potential of available water resources, and the government development plan in the area. An informant added that identifying present and future demand could help to allocate water resources for domestic, livestock, environment, agriculture, and industry, and also for catchment-based water allocation. Another key informant (KI-26) mentioned that the RVBDO was playing its role by working with respective Districts’ implementing agencies on conservation activities such as terrace and watershed development at the upper catchment to protect Lake Dembel. The informant also stated that RVBDO was educating/consulting individuals and companies polluting water resources although taking measures was not its mandate.

Regional water sectors such as Water and Energy, Agriculture, and Environmental Protection have also tried to exchange data and coordinate horizontally at the District level. They were exerting efforts to contribute to improved water governance in terms of awareness creation, capacity-building training, supporting irrigation equipment, and providing extension services through Development Agents (DA’s) at the Kebele level. One of the key informants (KI-11) mentioned that:

*Government institutions have played roles in irrigation water supply, and conservation activities by building a check dam at the upper catchment, mobilizing the community to plant grasses and trees to protect siltation from Dembel Lake, developing and maintaining irrigation schemes, registering and organizing IWUAs, providing irrigation water services, supporting women and economically poor people during IWUAs registration by providing pump, motor, and technical training and advice for farmers.*”

Data and information obtained for this study shows that public institutions have supported Irrigation Water User Associations to enhance their capacity to manage irrigation schemes and use the resources in a sustainable manner. Data obtained from participants of focus group discussion (FGD1) shows that public institutions have attempted to improve the efficiency of irrigation water uses (i.e. water saving, protecting wastage, implementing crop-water requirement technique, etc) by enhancing the awareness of the local water users to bring behavioral changes and improve water use practices. The discussants explained that institutions have contributed in terms of supplying irrigation equipment, providing technical advice related to cropping and water use, and mobilizing the local community to protect the environment in the CRV. The data indicates that the institutions conducted various capacity-building training, monitoring, and evaluation of the implementation of water sector policy and strategy, supporting IWUAs to strengthen community-based institutions for better water resources governance.

Even though these public institutions have played roles in improving water resources governance in the CRV, this study pointed out some limitations of the institutions. These institutions were not coordinated and systematically not managing the resources by sharing roles and responsibilities. For instance, RVBDO was educating polluters while the Environmental Protection Authority (EPA) was mandated to take measures on polluters. However, there was no clear linkage between these two organizations to exchange data to make informed decisions. One of the key informants (KI-8) stated that:

“ *There is a license called the so-called ‘treat and release water license’. The license is given by the Regional Water and Energy Bureau (WEB). Any company that has this type of license must treat wastewater before releasing it to water bodies. Due to lack of strong coordination between WEB and EPA, and absence of enforcement mechanisms, significant measures were not taken on polluters.*”

Another observation of this study was related to the capacity of public institutions which were supposed to play a great role in improving water governance in the study area. Data and information obtained for this study show that public institutions operating at lower levels (at Kebele and District) were not fully capacitated by human resources skills, required facilities, logistics, budget, and decision-making mandates. One of the key informants (KI-13) mentioned that “ *Licenses were provided by Region or Zone where relatively better capacity exists based on the study conducted by District experts where capacity is relatively low.*” Public institutions at regional and zonal levels have not played expected roles in terms of capacitating lower-level institutions by knowledgeable experts, finance, technology, logistics, etc to improve water resources governance. In addition, water sectors at the District level were not empowered to take corrective measures for non-compliance with water rules and regulations in the CRV of Ethiopia.

Respective sectors are expected to conduct a detailed study before providing licenses related to water projects. In the case of CRV, water projects were designed either at the Zone or Region level without detailed study. One of the key informants (KI-13) mentioned that:

“ *Decisions such as license for projects are made at Region and Zone. The projects are not supported by a detailed study that shows the availability of water (what quantity of water is available, how much quantity of water the project needs, how much quantity of water remains for the environment, etc) are not studied by project owners either government or private. Similarly, the Agriculture Office organizes IWUAs by simply observing the availability of water resources (without a detailed study of the availability of water resources, environmental flow requirements, irrigable land size and volume of water demanded, amount of water required for irrigation discharging, etc).*”

Moreover, projects designed at the upper catchment were not supported by a detailed study such as the amount of water discharged throughout the year, the number of populations whose livelihoods depend on the river at the lower catchment, the quantity of water could be diverted at upper and need to flow for the lower catchment. Irrigation projects at the upper catchment were implemented without considering these data and information.

As a result, water resource degradation still continued in the study area. For instance, over-abstraction and waste of water resources were continued. Irrigation water practices were not in the saving approach on the field, and users were not protecting the resource from exploitation. The lack of enforcement mechanisms and the absence of clear roles and responsibilities of water sector institutions were among the causes of the failure to implement the promulgated and decreed policies and regulations. Water resources management policies, rules, and regulations were not adequately implemented. As a result, the behavior and misuse of water users were not significantly changed in the CRV. One of the key informants of the study (KI-22) stated that:

*“The behavior of water users' was not changed, and a majority of users did not understand the impact of current water over-abstraction and malpractices because a majority of water users and implementing agencies did not understand well the severe water scarcity unfolding in the area.”*

According to data and information obtained, a lack of monitoring and evaluation system, an absence of institutional performance evaluation, a lack of coordination and integration among water sectors, a low level of public organizations’ capacity and community awareness, and a lack of implementation capacity at a local level were the factors affecting the effectiveness of public institutions in the CRV. One of the key informants (KI-23) mentioned that *low policy awareness of implementers and lack of integrated plans and actions among water sectors contributed to the inefficiency of formal institutions in water governance in the CRV of Ethiopia.*

Data obtained from the focus group discussions (FGD2) indicated that *there was a committee established from various sectors to mobilize the community for specific water resources management and environmental conservation activities in the CRV.* However, the efforts were not systematically assessed, and its implementation lacked continuity and consistency. Participants have also addressed that insufficient budget and logistics to reach farmers in remote areas, and lack of knowledgeable experts on water resources management and governance hindered the role of these institutions in water resources governance water in the study area.

### Role of IWUAs, development partners and NGOs in water governance in the CRV

In addition to government institutions, there are community-based institutions that have developed local rules/agreements by members of the IWUAs and are involved in water governance in the CRV of Ethiopia. Informants (e.g. KI-29 and KI-30) stated that IWUAs have contributed by developing local rules which address issues such as an irrigation water distribution schedule, sanction on non-compliance, annual fee contribution, conflict resolution mechanisms, and protecting water and scheme equipment from wastage and damages.

Participants of focus group discussion (FGD2) discussed that according to the IWUAs’ rules, any member of the IWUAs who violates the local regulations would be sanctioned according to their agreements. According to the discussants explanation, members of the IWUAs were expected to contribute to the sustainability of the irrigation system by developing and cleaning irrigation canals, developing soil and water conservation terraces, and removing harmful plants. In this regard, community members and the IWUAs committee have attempted to play roles to minimize irrigation water wastage and reduce conflicts in the CRV. However, the behavior and practices of water users were not significantly changed in terms of protecting and saving water resources in the study area.

The major factors attributed to the low performance of IWUAs in terms of improving water resources governance in the CRV were related to financial limitations, skill gaps, and low implementation capacity. For instance, the report of the Agriculture Office of Adami Tullu Jido Kombolcha District indicates that out of 70 active IWUAs in the District, about 87% of them have no offices and documentation facilities, 55% have no local regulations, and 19% are not legally registered and certified ( Adami Tullu Jido Kombolcha District Agricultural Office, 2020). Moreover, the local regulations decreed by the associations were not adequately practiced among irrigation water users in the study area. Information obtained from key informant interviews (e.g. KI-28) indicates that the main factors attributed to the inefficiency of IWUAs were the failure of public institutions' support and lack of continuous monitoring and evaluation systems, lack of capacity, low financial and technical capacity to undertake scheme operation and maintenance. Participants of the focus group discussion (FGD-3) also mentioned that lack of awareness and mistrust among some members, particularly on financial management, were factors attributed to the inefficiency of the associations in the study area.

In addition to IWUAs, there were also various development partners and NGOs who were exerting their efforts to improve water resources governance in the CRV of Ethiopia. For instance, Farm Africa, International Water Management Institute, Sustainable Environment and Development Agency, SOS Sahel Ethiopia, and Population and Health Consortium Ethiopia have been playing roles in terms of awareness creation on the effects of cutting trees and high water abstraction on irrigation sustainability, capacity-building training, and providing water-saving technologies, e.g. pumps, promoting catchment management practices in the CRV of Ethiopia. The main purpose of their engagement was to protect Lakes Dembel, Langano, and Abijata-Shalla which were under pressure due degradation of natural resources at the upper and middle catchments. The degradation was caused by soil erosion (siltation), excessive chemical use in irrigation, population pressure, and high-density rainfall (erratic nature). Moreover, Wetland International was also working on watershed management at the upper catchment, buffer zone development, and wetland management in the study area.

### The role of informal institutions in water resources governance in the CRV

In addition to formal institutions, there were also informal institutions contributing to water resources governance in the CRV of Ethiopia. When disputes and conflicts occurred because of disagreements between water users, elders mediated and resolved the disputes at the local level. There was a group of elders – very respected and known individuals, the so-called ‘Tulama’, who could resolve the disputes, particularly in Dugda Woreda. According to the statement of a key informant (KI-13), “Any individual who ignores the decision made by the ‘Tulama’ would be socially sanctioned.” The ‘Tulama’ is a broad informal institution that manages various issues including disagreements between individuals on irrigation water use in Dugda Woreda. The role of this institution in resolving problems was great, and it is also recognized by the District Administration.

Traditionally, the community members were protecting water sources from pollution and maintaining their cleanliness. One of the key informants (KI-5) stated that there were social norms that encouraged the purity of water sources by saying “ *Malkaa gubbaatti hin fincaa’iin ykn hin bobba’iin*”, which means never urine or defecate at the head of water sources. Similarly, another key informant (KI-13) mentioned that “ *the communities were protecting lands surrounding water bodies by protecting lands from cultivation/farming, which was previously covered by water bodies, instead they have planted trees.*”

Sources of this study indicate that the opportunity of implementing informal rules is greater than the governments in some areas because community members trust the local elders more than others. One of the key informants (KI-29) mentioned that “ *Community members have contributed a lot to the environment by involving forestation, watershed management, soil and water conservation, optimizing fertilizers, etc.*” Moreover, another source of this study (KI-19) pointed out that the local social norms, traditions, customs, and practices were used as a source for IWUA's rules and agreements. The informant added that informal institutions could be more fruitful if the government institutions supported them in terms of providing extension services and technical advice without interfering in their local agreements.

Even though informal institutions have played a great role in the governance of water resources in the CRV, various factors have hindered its effectiveness. One of the major factors was the lack of strong support from policy direction. The majority of water-related rules and regulations decreed by the country have not addressed the roles and means of informal institutions' engagement in water resources governance. In the Ethiopian Water Sector Strategy ( FDRE 2001), there is an article that promotes the involvement of religious and customary organizations in water source selection, public awareness, and environmental education. However, there were no clear enforcement mechanisms devised to implement the provisions in practice. In other water resources policy documents, the issue was partially or completely untouched.

On the other hand, the existing traditional practices and norms that had been used in earlier times in water governance at the local level were replaced with formal institutions such as IWUAs and farmers’ cooperatives. One of the key informants (KI-13) mentioned that in earlier times elders were educating children and younger by saying that “Making open defecation at the head of water sources is forbidden and unethical.” This was how the community members were protecting water sources traditionally from pollution in the area. However, nowadays such traditional advice and practices are not observed in a wider manner in the community. The informant associated this with the effect of the modern supply of water resources by pipelines, pumps, and water points for drinking water as it minimized interaction with open water sources partially or completely. In addition, informal institutions may lack data so that formal institutions may have to make informed decisions. The informant added that in recent times farmers were cultivating closer to water bodies due to population pressures and economic factors such as meeting current production and benefits.

Moreover, these informal institutions lacked capacity-building support to play more roles in the governance of water resources in the CRV of Ethiopia. One of the key informants (KI-31) mentioned that District and kebele-level public institutions and other partners had not provided sufficient support and capacitated them to enhance the capacity of informal institutions at the local level. The informant addresses that giving priority to formal institutions and losing attention to informal institutions from public organizations negatively affected the role of informal institutions in water resources governance in the study area.

### Interplay between formal and informal institutions in water governance in the CRV

Understanding the types of institutions and their interplay can indicate how the resource users engage in their common-pool resources governance ( Etiegni *et al.* 2017). In the case of CRV, rules decreed by the government and the traditional practices advocated by elders such as ‘Tulama’ in Dugda District were similar and overlapped in many aspects of the governance of water resources. For instance, both formal and informal institutions promote efficient water use and equitable distribution of water resources among water users. Both institutions also discourage the over-abstraction of water resources and impose sanctions on non-compliance. These institutions were interacting with one another to improve water resources governance in the area.

Although the rules and procedures of formal and traditional practices of informal institutions involved in water resources governance were supporting one another, the practices of water users contradicted the rules and traditional customs. Both formal and informal institutions promote saving irrigation water use, environmental conservation, and equitable and fair distribution of irrigation water. However, irrigation water users were practicing over-abstraction, flooding excessive quantities of water on their farms, and degrading water resources in the CRV of Ethiopia. One of the key informants (KI-11) mentioned this:

*“Farmers assume that they are more productive by flooding more water on their farming, which is opposite to what public (formal) institutions were advocating in that excessive irrigation of crops negatively affects the quantity of water, increases the cost of fuels of pumping, causes erosion of soil and agro-chemical inputs, reduce productivity, consume more time of the farmers, disturb the schedule of other irrigators and negatively affects water efficiency.”*

On the other hand, the local community believes that every member of the community should benefit from the available water resources and projects related to water in the area. One of the key informants (KI-5) states that “ *The society in the CRV believes that ‘water is for all*’, ‘ *malkaan kan hundaati*’ *. The government constructed irrigation schemes benefiting farmers who have irrigation land in the irrigation compound. Farmers who do not have irrigation land in the identified irrigation compound were not benefiting from the schemes though the budget used to construct the schemes was public funds, which should equally benefit all the community members in the area.*” In this case, the public institutions' action, which is benefiting those who have a plot of land in an irrigation compound, contradicts the society’s belief that ‘ *water is for all*’. Hence, there should be some mechanisms that benefit all community members either devising rotation or cost recovery of the public budget that was used for the scheme construction. If the gap in the economy between the scheme users and non-users is widening, it may be a source of conflicts in the future.

In general, various organizations were involved in different aspects of water resource matters such as its management and uses in the CRV. These organizations include government organizations (both federal and regional States), community water user associations, individual water users (farmers and private companies), development organizations, and NGOs. However, interlinks among the institutions were weak to positively influence one another to improve water resources governance in the CRV of Ethiopia. For instance, to improve water resources governance and other resources in the area, committees were organized from different water sectors at District levels. However, the established committee was not inclusive; the committee did not include representatives from the community, development partners, NGOs, individual investors, fishery associations, and other private companies who were very decisive for sustainable water resources in the study area.

## Discussion

### Role of formal institutions in water governance in the CRV of Ethiopia

The Constitution of the Federal Democratic Republic of Ethiopia recognizes the need to enact laws for the utilization and conservation of land and other natural resources. Following the Constitution, several water-related policies, rules, and regulations were promulgated and decreed by federal and regional governments. These rules and regulations advocate the necessity of building and strengthening water sector institutions in terms of facilities, human resources, finance, system, and institutional stability. The rules and regulations also promote users-based-management of irrigation systems, appropriate institutional structures, as well as decentralization to the lowest level of governance for better and more efficient management of water resources. Following the rules and regulations, public institutions were established and arranged to implement and enforce the policies, rules, and regulations at all scales.

The result of this study revealed that public institutions have attempted to play their roles to improve water resources governance through awareness creation about water saving and environmental protection, supporting irrigation equipment, and providing technical advice to irrigator farmers on how to use scarce water resources in a sustainable manner. In addition, the government organizations have conducted various capacity-building training, monitoring and evaluation, and support local institutions to strengthen community-level institutions for better water resources governance. The institutions have also attempted to decentralize water resources governance to the lower administration levels.

Although public institutions have played immense roles in improving water governance in the study area, the efforts were setback by different causes. For instance, the absence of clear roles and responsibilities for every actor, lack of harmonized integration, lack of skilled human resources at lower administrative levels, insufficient budget and logistics, and lack of full decentralization were the majors. The result agreed with the arguments reported in previous studies which state that lack of clarity on roles and responsibilities, and poor implementation capacity at a lower scale can constrain the success of decentralization in water resources governance ( Stoa 2014; Hegga *et al.* 2020).

The lower level of water institutions in the CRV lacked the capacity to make effective the rules and regulations of water resources management. The institutions lacked skilled experts to make assessments of water resources availability and collect relevant data, process data and disseminate information. At a lower administrative level, funds for operation and maintenance were not available in the study area. The existing local water institutions were not capacitated with the required facilities to identify specific characteristics and requirements regarding the water resources situation and devise operational strategies to implement water rules and regulations in the CRV of Ethiopia.

Water sector rules and regulations promulgated at different levels set obligations that irrigators must comply with when using water resources. For instance: efficiency of water use, preventing wastage and pollution of water, fairness and equity in water distribution, and protecting and administering irrigation and drainage systems can be mentioned as an example. However, these commitments were not attained in the CRV of Ethiopia. The higher water demand and inefficient use of water resources for irrigation were leading to over-abstraction and water resource degradation in the study area. Moreover, excessive water losses, discharging of untreated wastewater to water bodies, overuse of agrochemical inputs, and farming buffer zone areas were continued by water users in the CRV, contrary to the decreed rules and regulations. Public institutions could not place an accountability system on non-compliance by bringing them to the court system or other comparable mechanisms. These limitations, exacerbated by water hyacinth invasion, put the resource under serious stress and a fragile situation. This implies that public institutions engaged in water resources governance have not achieved results associated with their roles in the study area.

In addition to public organizations, there were also other institutions such as IWUAs, Development Partners, and NGOs engaged in water resources governance in the CRV of Ethiopia. IWUAs have attempted to contribute to water resources governance in irrigation water distribution, conflict resolution, protecting irrigation schemes and equipment from damage, and collecting annual fees for maintenance and operation. In addition, various development partners and NGOs have also exerted their efforts via awareness creation, capacity building training, watershed management, wetland management, and providing irrigation technologies to improve water resources governance in the CRV of Ethiopia. These development partners and NGOs also contributed to activities such as integrated natural resources management (reforestation, conservation, etc).

However, the efforts of these institutions were not systematically integrated and coordinated. The efforts of these organizations were not interlinked with public institutions and with other similar institutions to boost the contribution of the institutions. There was no strong platform that could bring all actors to plan, act, and evaluate together to intervene in the problem systematically. In addition, many works have been done by different development partners and NGOs were slid back due to a lack of an institutionalized system that sustains the initiatives designed by development partners and NGOs when their projects were phased out.

### Role of informal institutions in water governance in the CRV

In addition to formal institutions, informal institutions such as local institutions, traditional practices, customs, and norms have been positively contributing to water resources governance in the CRV. For instance, traditional practices of the so-called ‘Tulama’ in Dugda District have been contributing positively to water resources governance in terms of equitable distribution of irrigation water, and resolving disputes and conflicts. A ‘Tulama’ is a local institution established by a group of respected and well-known elders who resolve various disputes including water conflicts in the Dugda District. These respected elders mediate among disputants after understanding the cause of their conflicts. The ‘Tulama’ made a decision that the disputants would accept. If any of the disputant parties refused the decision made by ‘Tulama’, the individual/s would be socially sanctioned. The District Administration has also recognized the role of ‘Tulama’ in conflict resolution. This type of institution was also reported from a study conducted in the Borena Zone of Oromia which indicates ‘Abbaa konfi’ who first excavated the pond and the ponds were administered by respected local elders ( Edossa *et al.* 2007). There were also the community’s customary practices such as developing terraces for soil and water conservation, planting trees in their home garden, and arranging irrigation schedules considering priority for women and elders in the study area.

Several studies also show that informal institutions could play significant roles in natural resource governance in many parts of the world ( Pellowe and Leslie 2020). For instance, a study conducted in northern Ethiopia, in Zimbabwe, and in India reported that informal institutions have contributed to positive outcomes in resource governance including water resources ( Degefa 2010; Nhundu *et al.* 2015; Haider 2017). This positive influence of informal institutions on resource governance implies that the rural community set up in most developing countries including Ethiopia has greater values for local leaders and respected individuals such as elders and religious leaders, and they were playing great roles in resource governance.

Although informal institutions were contributing to improving water resources governance in the CRV, different factors were setting back its effectiveness in the area. The major factors attributing to the lower efficiency of these institutions in the study area were: a lack of technical and financial capacity and a lack of organized working environment and facilities can be mentioned as an example. Similar to this finding Tortajada (2010a) mentioned that even though informal institutions have played key roles in the governance of commons like water bodies, many of these institutions were challenged due to a lack of support, limitation of funds, and lack of capacities. This study also revealed that the informal institutions were hampered due to a lack of policy support and limited support from public agencies. Previous studies argued that informal institutions can contribute positively to resource governance if they are considered during policy formulation, and interlinked with the existing formal governance system ( Anaba 2016). Hence, it is important to consider the role of informal institutions when policies, rules and regulations are drafted, as well as when public agencies are established for the management of water resources.

### Interplay between institutions in water governance in the CRV

Understanding the types of institutions and their interplay can indicate how the resource users engage in their common-pool resources governance ( Ostrom 2011; Etiegni *et al.* 2017). There were instances where institutions conflicted or overlapped with one another. These conflicts were the results of incongruence between the objectives of formal institutions and the practices of water users or the rules of informal institutions ( Pellowe and Leslie 2020). In the case of CRV, the objectives and roles of both formal and informal institutions were overlapping in many aspects. For instance, monitoring and evaluation techniques, conflict resolution mechanisms, means of irrigation water distribution, and conservation activities of water resources were some of the overlapping roles and operating mechanisms of formal and informal institutions in water governance efforts in the study area.

On the other hand, the interplay between formal and informal institutions was weak and not systematically coordinated. Water institutions engaged in the CRV were not well interlinked horizontally and vertically at all scales. The organizations lacked an integrated plan and missed the opportunity of collaboratively operating to efficiently use the available resources that enable better water sector performance. Organizations engaged in water resources governance were failed to engage and coordinate all relevant water-related institutions operating in the study area, and were unable to establish a common stakeholders' platform. Fragmented efforts were the common feature of institutions in water resources governance in the study area. This finding is similar to Jacobi *et al.*(2014) reports, which addressed that major challenges related to water institutions are a lack of integrated planning, a lack of coordination among key actors, and missing management mechanisms that may fit local conditions.

Moreover, the interplay between water institutions engaged at the federal, regional, Woreda, and local level were not strong enough to advance the efforts towards improving water resources governance in the CRV of Ethiopia. Hence, identifying the types of institutions involved in the governance of water resources, understanding how the existing institutions interact, and considering an integrated view of these institutions to shape water users' behavior is essential to improve water resources governance and sustainable uses of water resources, particularly in the CRV of Ethiopia.

## Conclusion and Recommendation

There were various formal and informal institutions engaged in water resources governance in the CRV of Ethiopia. The formal institutions have played significant roles in terms of providing training for water users and agencies, supporting irrigation equipment, and providing technical advice to irrigators about cropping, water use, and how to protect the environment. In a similar manner, informal institutions were also contributing to water resources governance in terms of awareness creation, conflict resolution, and environmental conservation in the CRV. The roles of these institutions overlapped in many aspects. However, the interplay between the institutions was weak in water resources governance in the CRV. There were also instances where both types of institutions failed to harmonize in improving water resource governance due to the absence of equal attention for both types of institutions; particularly, a lack of clear supporting policy for informal institutions. Lack of strong monitoring and evaluation, lack of incentives, and low level of capacity at lower administrative scales were the main factors contributing to the low performance of water institutions in the study area. These limitations were also associated with weak interlinks between both formal and informal institutions, which emanated from a lack of consideration in policy formulation. As a result, actions undertaken via both institutions did not significantly change the behavior and practices of water users, and were unable to improve water resources governance in the CRV. As a consequence, water resources degradation was continued in the study area, which was in turn affecting the livelihoods of the community, stressing the sustainability of irrigation activities, and degrading the environment. Hence, revisiting how formal and informal institutions could be synergistically embedded, and reconsidering the role of informal institutions in water policy, rules, and regulations are essential. It is also very important to reconsider customary and traditional practices, and socially constructed values, and respected elders could engage in managing water resources. Moreover, developing strategies and techniques to strengthen the interplay among all institutions involved in water governance at different scales, meaningful decentralization and key stakeholders engagement, and horizontal and vertical institutional integrations of water sectors need greater attention to enhance the role of institutions, and to improve water governance in the country in general and in the CRV in particular.

## Data Availability

OSF: Underlying data for “The Role and Interplay of Institutions in Water Governance in the Central Rift Valley of Ethiopia” OSF Storage in The Role and Interplay of Institutions. https://doi.org/10.17605/OSF.IO/AP8HT ( Jibat *et al.*, 2023) This project contains the following underlying data:
- Transcribed data for the role of institutions OSF: Underlying data for “The Role and Interplay of Institutions in Water Governance in the Central Rift Valley of Ethiopia” OSF Storage in The Role and Interplay of Institutions. https://doi.org/10.17605/OSF.IO/AP8HT ( Jibat *et al.*, 2023) This project contains the following extended data:
- Interview guide for key informant interviews and focus group discussions Data are available under the terms of the Creative Commons Attribution 4.0 International license (CC-BY 4.0). “Adami Tullu Jido Kombolcha District Agricultural Office Annual Report (2020)” is an unpublished hardcopy report document that the corresponding author (EJ) obtained by providing a support letter from the university. A reader or reviewer may apply for access to the report by contacting the Adami Tullu Jido Kombolcha District Agricultural Office via phone (+251)0464413479.
