# Supplementary material for: The role and interplay of institutions in water governance in the Central Rift Valley of Ethiopia
Source: F1000Res. 2024 Apr 8;12:1434. Originally published 2023 Nov 6. [Version 2] doi: 10.12688/f1000research.138939.2 (PMC11140302; doi:10.12688/f1000research.138939.2)
Supplement: Peer review history

# Reviewer report: Reviewer response for version 2

**DOI:** 10.5256/f1000research.163800.r264126

**Published:** 2024-05-30

**Authors:** Samwel J Kabote

**Affiliations:** 1 Department of Development Studies, Sokoine University of Agriculture, Morogoro, Tanzania

I have no further comments. My previous comments have been considered.

Is the work clearly and accurately presented and does it cite the current literature?

Yes

If applicable, is the statistical analysis and its interpretation appropriate?

Not applicable

Are all the source data underlying the results available to ensure full reproducibility?

Yes

Is the study design appropriate and is the work technically sound?

Yes

Are the conclusions drawn adequately supported by the results?

Yes

Are sufficient details of methods and analysis provided to allow replication by others?

Yes

Reviewer Expertise:

Development Studies with research interest in water governance

I confirm that I have read this submission and believe that I have an appropriate level of expertise to confirm that it is of an acceptable scientific standard.

# Reviewer report: Reviewer response for version 2

**DOI:** 10.5256/f1000research.163800.r264125

**Published:** 2024-04-30

**Authors:** Mekonnen Bogale

**Affiliations:** 1 Jimma University, Jimma, Ethiopia

Thank you for communicating me with improved vision of the manuscripts after the author has incorporating the comments I provided. I saw the improved one and I am happy with the authors revisions.

Is the work clearly and accurately presented and does it cite the current literature?

Yes

If applicable, is the statistical analysis and its interpretation appropriate?

Yes

Are all the source data underlying the results available to ensure full reproducibility?

Yes

Is the study design appropriate and is the work technically sound?

Yes

Are the conclusions drawn adequately supported by the results?

Yes

Are sufficient details of methods and analysis provided to allow replication by others?

Yes

Reviewer Expertise:

my area of research is management and governance

I confirm that I have read this submission and believe that I have an appropriate level of expertise to confirm that it is of an acceptable scientific standard.

# Reviewer report: Reviewer response for version 1

**DOI:** 10.5256/f1000research.152175.r239502

**Published:** 2024-03-05

**Authors:** Mekonnen Bogale

**Affiliations:** 1 Jimma University, Jimma, Ethiopia

The article examines the role of institutions in ensuring water governance in Ethiopia, with a focus on the Central Refit Valley.  The premise of the article is that institutions' interactive intervention can be crucial in coordinating the efficient management and responsible use of natural resources without going overboard with exploitation.  Laws, regulations, and organizational structures that allow, prohibit, or control human behavior are known as institutions. Methodologically, qualitative research approach was used, which I feel the correct choice to examine such types of research.  

 The study's findings showed that there was little to no impact of informal institutions on formal institutions or vice versa, and that these institutions were unable to alter the behavior of water management within the Central  Rift Valley. Unclear roles and responsibilities among them, insignificant power decentralization, lack of integrating policies and other enforcement measures are issues that create loose performance linkage among the institutions. 

 The study is helpful in informing those who develop water management policies and legal frameworks about the importance of taking into account the integrative performance of various institutions, both formal and informal, to ensure water utilization governance in the particular context.  It will also help academics gain an empirical understanding of the problems.  

 With all the above general qualities of the article, it has the following minor and major flaws to be improved .

 Minor comments 

- The novelty of the study is not mentioned. The abstract will be better if the authors show the uniqueness of the study in a few words at the end of the abstract.
- Keywords are not arranged in alphabetical order
- The definition of water management in the first line of paragraph two of the introduction part needs rewriting in a way that shows 'water management' is a process 

Major comments 

- The knowledge gap this study intended to fill is not mentioned at the end of the introduction part.  Please revisit and clearly show this to the readers or scientific community. It could be methodological, conceptual, or study period gaps that you identified from the prior studies to fill as a gap. 
- You should review theoretical literatures that emphasize how formal and informal institutions interact to ensure resource governance in order to provide a solid foundation for your article..  
- In the research design part of the study, the authors have mentioned survey design and sample survey to generalize about population based on the sample. Do you think that such a design is appropriate for your study, which applied Key informant interviewand focus group discussion to collect qualitative information? The key informants you mentioned as data sources are not coincident with this frame. Please revisit and correct this.  
-  Line 6/7 of sampling technique paragraph is about sampling frame of farmers, which is best fit for selecting representative farmers for statistical analysis using systematic sampling technique.  However, yours is qualitative which was relied on households/farmers which were purposely selected for Focus Group Discussion/Key informant interview. Please check this also.  As a result Paragraphs 1, 2, and 3 of the sampling technique part has been abet confusing and need improvement and alignment with each other.
- The purposively selected Kebeles from each Woreda   (districts) are not mentioned.
- The first part of the discussion part (Role of formal institutions in water governance in the CRV of Ethiopia) lacks supportive latest empirical studies conducted in similar settings. You have used only one. Please revisit and support this by similar empirical studies.

Is the work clearly and accurately presented and does it cite the current literature?

Yes

If applicable, is the statistical analysis and its interpretation appropriate?

Yes

Are all the source data underlying the results available to ensure full reproducibility?

Yes

Is the study design appropriate and is the work technically sound?

Yes

Are the conclusions drawn adequately supported by the results?

Yes

Are sufficient details of methods and analysis provided to allow replication by others?

Yes

Reviewer Expertise:

my area of research is management and governance

I confirm that I have read this submission and believe that I have an appropriate level of expertise to confirm that it is of an acceptable scientific standard, however I have significant reservations, as outlined above.

# Reviewer report: Reviewer response for version 1

**DOI:** 10.5256/f1000research.152175.r227329

**Published:** 2024-02-15

**Authors:** Samwel J Kabote

**Affiliations:** 1 Department of Development Studies, Sokoine University of Agriculture, Morogoro, Tanzania

The article dwell on a critical and pressing issue of water governance. It is well written but needs minor improvement in the following areas:

 1. On page 4, the author should a bit improve description about context of the study area by including water bodies available in Ethiopia particularly in the Central Rift Valley of Ethiopia. 

 2. On page 7, third paragraph, it looks the author used secondary data. However, detailed information about sources of data, types of data and how data were analyzed is missing. This should be improved.

 3. When making quotation from a key informant, the author should indicate date, place of interview and title of the key informant 

 4. The sub-titles used in the results section and in the discussion section are almost similar. This brings confusion and so needs improvement to avoid repetition

Is the work clearly and accurately presented and does it cite the current literature?

Yes

If applicable, is the statistical analysis and its interpretation appropriate?

Not applicable

Are all the source data underlying the results available to ensure full reproducibility?

Yes

Is the study design appropriate and is the work technically sound?

Yes

Are the conclusions drawn adequately supported by the results?

Yes

Are sufficient details of methods and analysis provided to allow replication by others?

Yes

Reviewer Expertise:

Development Studies with research interest in water governance

I confirm that I have read this submission and believe that I have an appropriate level of expertise to confirm that it is of an acceptable scientific standard, however I have significant reservations, as outlined above.

# Response

**Published:** 2024-03-25

**Authors:** Endalew Jibat

**Affiliations:** Center for Environment and Dev, Addis Ababa University, Addis Ababa, Addis Ababa, Ethiopia

Amendments from Version 1

 We added a definition of water management. The order of keywords was also arranged in chronological order. We added institutional theories and frameworks used. The study considered various theories and approaches and used some components to analyze the roles and interplay of water institutions in the study area. We added water resources available in the CRV of Ethiopia.
